# Role of Innate Immunity in Pediatric Post-transplant Idiopathic Liver Fibrosis

**DOI:** 10.3389/fimmu.2020.02111

**Published:** 2020-10-22

**Authors:** Yue Wu, Mingzhu Huang, Haojie Sun, Xiying Zhou, Ruoqiao Zhou, Guangxiang Gu, Qiang Xia

**Affiliations:** Department of Liver Surgery, Renji Hospital, Affiliated to Shanghai Jiao Tong University School of Medicine, Shanghai, China

**Keywords:** liver fibrosis, pediatric liver transplantation, innate immunity, NK cells activity, dendritic cell (DC)

## Abstract

Pediatric post-transplant idiopathic liver fibrosis is an unexplained graft fibrosis that occurs in symptom-free children without acute rejection and surgical complications. Despite a lack of consensus on the subject, the development of pediatric post-transplant idiopathic liver fibrosis is believed to be the result of multiple potential factors, including ischemia-reperfusion injury, allogeneic acute and chronic rejection, viral hepatitis recurrence, opportunistic infection, and drug-induced liver damage. Among them, there is growing evidence that innate immunity may also have a unique role in this progression. This study reviews the features of pediatric post-transplant idiopathic liver fibrosis and discusses current studies illustrating the potential mechanisms of liver allograft tolerance induced by intrahepatic innate immunity, the role of components including Toll-like receptors (TLRs), interferons (IFN), dendritic cells (DC), natural killer cells (NK cells), NKT cells, neutrophils, and Kupffer cells, as well as their possibly relevant role in the development of pediatric post-transplant idiopathic liver fibrosis.

## Introduction

Pediatric liver transplantation is one of the most effective choices of treatment for many advanced liver diseases in children. Most pediatric primary liver diseases can be cured through liver transplantation, however, the incidence of post-transplant idiopathic liver fibrosis is extremely high in this population (1–3). It was reported by Scheenstra that the prevalence of fibrosis increased from 34 to 48, 65, and 69% among 77 children (0.1–16.8 years old) in 1, 3, 5, and 10 years follow-up after liver transplantation, respectively (4). As liver fibrosis progresses to cirrhosis, the life quality of patients deteriorates gradually, whereas a long-term study by Venturi reported that liver function of 70% pediatric recipients with graft fibrosis has been maintained within an acceptable fluctuation range.

At present, it has been proved that innate immunity plays a crucial part in immune intolerance after graft transplantation and in liver fibrosis, while the detailed mechanism remains unclear in pediatric post-transplant idiopathic liver fibrosis. In this review, we discuss current studies, illustrating the potential mechanisms of liver allograft tolerance induced by intrahepatic innate immunity. We also examine the role of some essential components in innate immunity and its possible role in the development of pediatric post-transplant idiopathic liver fibrosis.

## Pediatric Post-Transplant Idiopathic Liver Fibrosis

Pediatric post-transplant idiopathic liver fibrosis is an unexplained graft fibrosis that occurs in symptom-free children recipients without acute rejection and surgical complications. Even though laboratory tests may suggest normal or subnormal liver function, fibrosis usually affects the life quality and long-term survival rate of recipients (5). Damaged hepatocytes stimulate and activate hepatic stellate cells (HSC) through two routes: (1) the release of damage-related reactive oxygen species and other fibrogenic substances; (2) the recruitment of immune cells which promote cytokines and chemokines to cause further collagen fiber deposition (6). The mutual stimulation between inflammation and profibrotic cells leads to a vicious circle of liver fibrosis. Compared with liver fibrosis in non-transplanted patients, the development of post-transplant liver fibrosis has potential factors, including ischemia-reperfusion injury, allogeneic acute and chronic rejection, viral hepatitis recurrence, opportunistic infection, and drug-induced liver damage, etc.

The traditional histologic scoring system for liver fibrosis does not effectively recognize the unique pattern of graft fibrosis. In addition to the METAVIR staging system and Ishak score which ignore the patterns of fibrosis in the pericellular region and around the central vein, a new staging system is required to detect the fibrosis level. It is found that pediatric post-transplant idiopathic liver fibrosis can be generally divided into three patterns: periportal, centrilobular, and perisinusoidal (7). Up till now, many multicenter studies have suggested that these histopathological changes reflect an active and sustained immune response which may involve a chronic ill defined immune mechanism (8). It has been reported that recipients with chronic hepatitis are more likely to develop periportal fibrosis after liver transplantation due to portal inflammation, interfacial hepatitis, and biliary ischemia possibly induced by ischemia-reperfusion injury (9, 10). Centrilobular fibrosis, which is defined as fibrosis around the central vein, may be the result of chronic rejection after liver transplantation (11). A study of 10 years follow-up in 80 pediatric patients, stated that the immune-mediated central perivenulitis is a risk factor for centrilobular fibrosis. This tends to be related to ductopenic chronic rejection (12). In some cases, antibody-mediated chronic rejection may facilitate the progression of perisinusoidal fibrosis (8, 13–16). However, fibrosis and inflammation were occasionally disconnected: some biopsies indicate mild to moderate fibrosis without inflammation, while others present inflammation with a low Ishak fibrosis score. No firm conclusions have yet been reached about the clinical prognosis of different fibrosis patterns, and there are gaps in current knowledge.

## Predominant Innate Immunity in The Liver

There are a large number of innate immune cells involved in immune recognition and response, including Kupffer cells, dendritic cells, natural killer cells, NKT cells, neutrophils, and so on. These innate immune cells are involved in constituting the hepatic immune microenvironment and activate adaptive immune responses (17). Other components also play roles in this process, such as pattern recognition receptors [e.g., Toll-like receptors (TLR) and humoral factors] (e.g., complement and IFN).

Compared to other solid organs, the innate immune system has a more specific significance in the liver. First, the liver is an organ with a double circulatory supply that receives blood from the portal vein and the hepatic artery. The liver must identify a large number of antigenic components from systemic blood circulation as well as the gastrointestinal tract. Innate immunity is the most important first-line defense for the body to resist and respond to invading pathogens, enabling it to fight them quickly early on, but without specific recognition of foreign antigens. Hepatic sinusoids, the unique structure of which enables blood to flow slowly, also prompt the immune cells and substances to fully present antigens and initiate other responses (17). Second, as a biosynthesis machine for protein, the liver is responsible for producing the majority of complement and pattern recognition receptors. Third, abundant Kupffer cells in the liver account for 80–90% total macrophages in people. NK and NKT cells are also abundant in the liver. There are also a variety of pattern recognition receptors including TLRs expressed in liver cells.

## Innate Immunity and Liver Fibrosis

### Toll-Like Receptors

Toll-like receptors (TLRs) are pathogen recognition receptors that bridge the innate immunity and adaptive immune response. At the time TLRs bind to appropriate ligands, the up-regulated cytokines and chemokines will induce dendritic cell maturation and adaptive immune activation.

**Figure 1:**
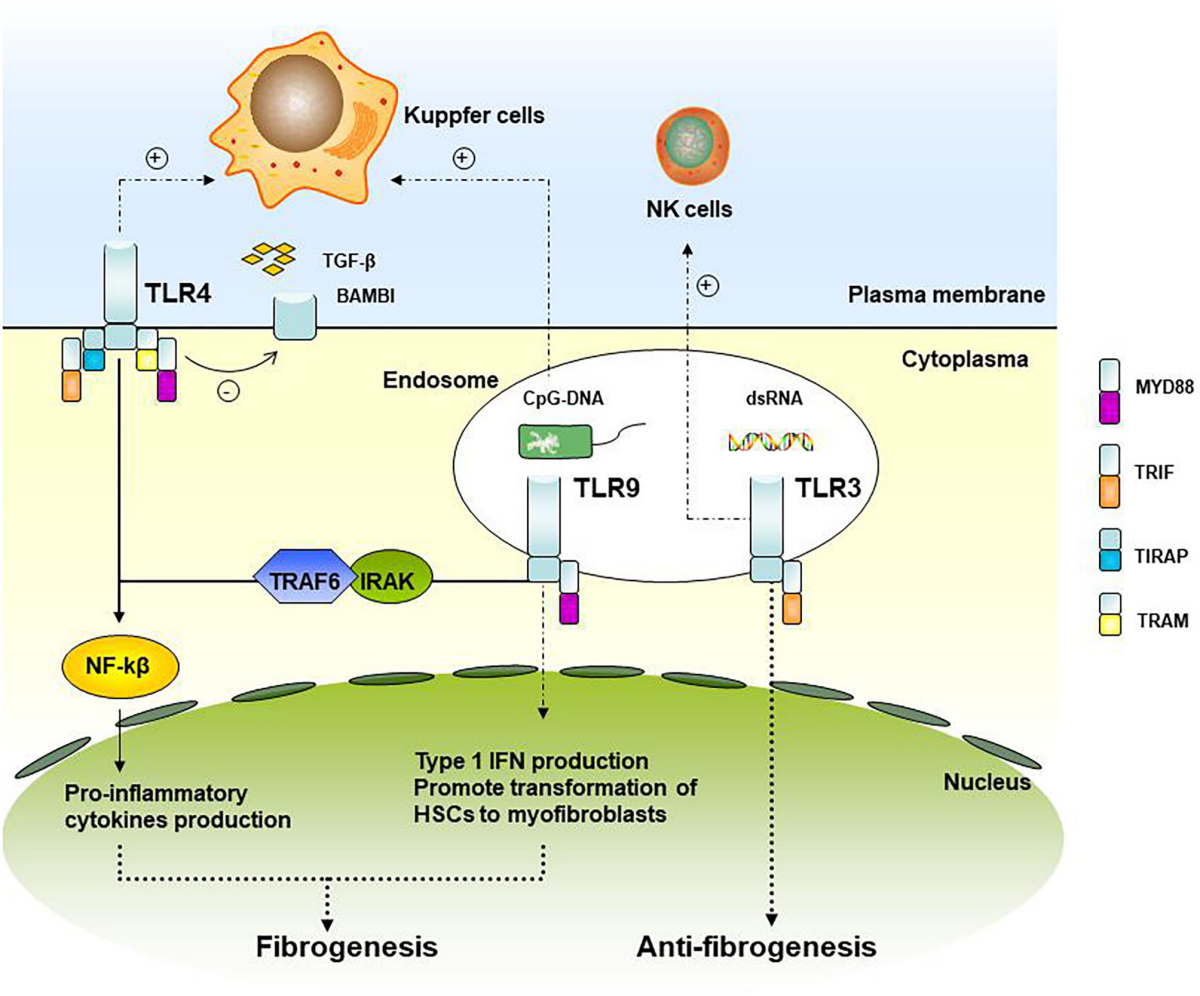
TLRs and its downstream signaling pathways associated with liver fibrosis. TLR4 and TLR9 signaling up-regulates the pro-inflammatory cytokines and type I IFN production and participating in graft fibrosis, while TLR3 is involved in the attenuation of fibrosis.

Ten functional types (TLR1–TLR10) have been found in humans, some of which indicate involvement in liver fibrosis progression (18). TLRs and the downstream signaling pathways associated with liver fibrosis are shown in Figure 1. TRL4 ligands do not directly stimulate HSC, TLR4 enhances the chemotaxis of Kupffer cells and activation of HSCs through down-regulated TGF-β mimic receptor Bambi (BMP and the activin membrane-bound inhibitor) by the NF-κB pathway, therefore promoting liver fibrosis (19). Experiments have demonstrated that TRL4 mutant mice were less likely to develop inflammation and fibrosis than TRL4 wild-type mice when all of them were processed with bile duct ligation, long-term CCl_4_, or thioacetamide-treated (19). In patients after liver transplant with acute rejection, TLR2 and TLR4 expression was found to increase circulating monocytes (20). Clinical research studies have also indicated that patients with TLR2 Arg753Gln homozygosity gain a higher average fibrosis score and that this homozygosity was associated with graft loss in HCV patients after liver transplantation, in which TLR2 signaling may play a role in accelerating the process of fibrosis (21).

As for TLR9, it recruits and activates the IRAK-TRAF6 complex through the binding of MyD88, promoting the activation of NF-kB and AP-1. TLR9 within HSCs can be combined with unmethylated CpG-DNA to promote the transformation of HSCs cells into myofibroblasts, a kind of fibrogenic cell (22). In liver fibrosis model mice with TLR9-knockout, the expression of a-SMA in the liver is down-regulated, indicating that TLR9 can promote liver fibrosis. Kupffer cells can also be activated by TLR9 recognition of CpG-DNA and secreting IL-1β, reducing the degradation of the extracellular matrix, and promoting liver fibrosis (23).

TLR3 is the only TLRs independent of MyD88. Wang et al. (24) showed that TLR3 ligand polyI: C can stimulate high-level expression of TLR3 in HSCs cells and production of IFN-β, inhibiting HSCs cell proliferation. At the same time, it enhanced the activation of NK cells, resulting in apoptosis of HSCs and attenuation of fibrosis.

TLR expression and the interaction between liver cells and immune cells are of great importance in graft tolerance. Therefore, manipulating the functional role of TLR signaling in transplantation may have the potential to be a unique therapeutic strategy.

### Interferons (IFN)

A variety of animal experiments have indicated that the up-regulation of IFN-α/β and IFN-γ can ameliorate liver fibrosis (25–28). Clinical studies among patients with chronic HCV infection including both virologic responder and non-responder have also shown that serum fibrotic markers and liver histology improve in those who were treated with IFN-α therapy (29–32), however, such therapy was less effective in patients with advanced fibrosis (29).

IFN-γ is a critical pro-inflammatory cytokine in the process of inflammation and acute graft rejection, and a well-known anti-fibrotic cytokine. IFN-γ is produced by NK cells and can induce HSC cell cycle arrest and apoptosis in a STAT1-dependent pathway to inhibit liver fibrosis (33). In a study done by Li et al. among advanced HBV patients with liver fibrosis, IFN-γ decreased significantly (34). According to a study by Ramalingam et al., blocking IFN-γ and IL-13 at the same time can offer more protection for patients with progressive fibrosis than blocking IL-13 alone (35).

IFN-λ plays an important role in the defense of viruses, bacteria, and fungi. Epithelial cells within the liver are uniquely IFN-λ sensitive due to selective IFNLR1 expression. IFN-λ also promotes inflammation and fibrosis in the liver. IFN-λ stimulates macrophages to become more cytotoxic and promotes their ability to phagocytosis. Meanwhile, interferon-stimulated genes (ISGs) are activated, and more pro-inflammatory cytokines and chemokines, such as CCR5 and CXCR3, are induced to enhance the migration of other immune cells and up-regulating their cytotoxicity. There are 4 members of IFN-λs in the human immune system. In particular, IFN-λ3 promotes the phenotype change of macrophage to be pro-inflammatory (36). In a study done involving non-alcoholic fatty liver disease patients, IFN-λ4 was found to be associated with liver fibrosis by modulation of innate immunity activation and necrotic inflammation (37). Macrophages are more crucial to the development of localized tissue inflammation in response to IFN-λ despite the fact that both dendritic cells and macrophages strongly respond to IFN-λ (38). Because IFN-λ signaling lasts longer (39) in chronic infections, prolonged immune activation can be achieved by its continuous expression.

### Dendritic Cells

Dendritic cells (DCs) are heterogeneous antigen-presenting cells and work to connect innate and adaptive immunity. The population of liver dendritic cells (LDCs) is of note, in that it has a low capacity to give an immunogenic response to antigen but tends to be tolerogenic (40). A classic study by Connolly et al. showed that in a mouse model treated by thioacetamide and leptin, LDCs potentially mediated the proinflammatory environment of liver fibrosis. They found that in the case of liver fibrosis, hepatic DCs would expand five times with CD11b^+^CD8^−^ myeloid DCs 20% higher, and B220+ plasmacytoid DCs 15% lower, which means LDCs acquired a subset of immunogenic phenotype and marked ability to initiate both innate and adaptive immunity mediated by TNF-α (41). Great importance should also be attached to the findings of other studies, which established that DCs from the fibrotic liver (FLDCs) directly activated HSCs. The FLDCs could induce a moderate increase of ICAM-1 before the activation of HSC. Additionally, CD40 expression could also be upregulated by FLDCs, which has something to do with the initiation of inflammatory signaling pathways in HSCs (41). It had been suggested that the modulation of LDCs may be a possible way to manipulate the inflammatory condition of the liver.

Henning et al. found that in non-alcoholic steatohepatitis (NASH) liver, LDCs play a regulatory role by clearing apoptotic cells and necrotic remnants. LDCs restrict the expansion of CD8+ T cells and the expression of Toll-like receptors and limit the production of cytokine in innate immune effector cells in NASH. As a result, the depletion of DC populations leads to delayed recovery from fibroplasia and intrahepatic inflammation (42).

After liver transplantation, the highly motile DCs would migrate to and from the transplanted liver. Demetris et al. assessed the situation of donor dendritic cell dissemination into recipient tissues and found that in all the records, residual donor DCs persisted irregularly in liver grafts including the portal tract area and terminal hepatic venules (43). When it comes to immunosuppressive therapy after liver transplantation, a study by Lee et al. demonstrated that intracellular processing events of antigens by DCs are partly inhibited by tacrolimus and cyclosporine A(CsA), two commonly used immunosuppressants post-surgery (44). These post-transplant LDCs alterations may lead to changes in the immune microenvironment in the liver allograft, and whether these changes could lead to post-transplant liver fibrosis or not requires further exploration.

### NK Cells

Compared to the NK cells in peripheral blood lymphocytes, the ratio of NK cells against all liver lymphocytes is much higher. In rat and human livers, about half of lymphocytes are NK cells (45). Using mouse models, two studies in 2006 first demonstrated the ability of NK cells in killing activated HSCs (25, 46). Other different studies in animal models (47–53) as well as in human patients (54, 55) have also confirmed this result. Further studies have suggested that the killing ability of NK cells is based on the activation status of HSCs: early activated (transitional) HSCs are killed by NK cells, while quiescent or fully activated HSCs are usually not. Research on the liver fibrosis of mice and HCV patients has shown that NK cells also produce IFN-γ, which inhibits liver fibrosis by inducing HSCs apoptosis. According to research done by Shi et al., compared with those from chronic hepatitis B patients, which enters HSCs to form emperipolesis and be apoptotic. Increased intrahepatic tumor-growth factor (TGF)-β plays a crucial role in this process (45). Activated HSCs impair the anti-fibrosis capacity of NK cells through a TGF-β-dependent way, which implies that NK cells and HSC cells interact with each other in the process of hepatic fibrosis. Recent evidence provided by Jeong et al. and some other research has suggested that the production of IFN-γ not only contributes significantly to inhibiting the activation of HSC cells but also enhances NK cells ability to kill activated HSCs by upregulating the expression of NKG2D and TRAIL on NK cells (45, 46). Other pathways, including the RAE-1/NKG2D, NKp46, and NKp30 pathways or the inhibitory NK receptor, also take part in NK cells' killing of HSCs.

A study by Obara et al. among post-liver transplant patients has shown that recipient-derived NK Cells produce IFN-γ after liver transplantation. By using up the NK cells and the concomitant decrease of IFNγ may contribute to prolonged graft survival. IFNγ is crucial in graft rejection and promoting tolerance induction (47). Hanvesakul et al. also found that regulating the mutual interaction between HLA-C and KIR is a new method to promote long-term graft and patient survival, to reveal the importance of NK cells in chronic rejection and liver fibrosis after transplantation (6). These studies have revealed that changes in NK cells and their related inflammatory factors as well as the vital role they play in post-transplant liver fibrosis. NK cells may also have an effect on post-transplant liver fibrosis among children and further studies are required to confirm their role in this specific situation.

NK cells in the liver have been divided into two subsets according to their unique expression: conventional NK (cNK) cells, which express CD49a–DX5+ and circulate freely, and liver-resident NK (lrNK) cells, which selectively reside in the sinusoids with CD49a+DX5– phenotype. Currently, there is a consensus that the expression of Tbet and Eomes is the best way to distinguish between these two subsets. Eomes-rich NK cells in the human liver do not recirculate but can be replenished from the circulation weeks after transplantation. Cytokines and surface markers also transform into the lrNK phenotype, which is contrary to Gordon and Daussy's findings in a mice model. Administering immunosuppression to recipients after transplantation may affect the recruitment of cNK to the liver and fill a resident niche. Despite many reports demonstrating the relation between NK cells and liver fibrosis, little is known about the role of liver-resident NK cells in fibrosis, as previous studies reckon the integral NK cells in the liver as research subjects, and result in crosstalk. For example, liver-resident NK cells are more efficient in producing IFNγ, TNFα, GM-CSF, and express in high TRAIL compared with cNK cells (48).

The distinct expression patterns of effector molecules subsets may play different roles in the pathogenesis of post-transplant liver fibrosis.

### NKT Cells

Natural killer T (NKT) cell populations are plentiful in the human liver. As a specialized subset of T cells that recognize lipids or glycolipid presented by CD1d rather than peptides, they express a large number of markers of receptors on both NK cells and T cells (49, 50). Natural killer T cells can rapidly secrete innumerable pro-inflammatory and anti-inflammatory cytokines (Th1 or Th2 type) and act on other innate cells as well as adaptive T and B cells (51). Many studies have considered that invariant Natural killer T (iNKT) cells play a part in hepatic fibrogenesis. Park et al. used animal experiments to show that iNKT cells can inhibit HSC activation by producing numerous anti-inflammatory cytokines (IFN-γ) as well as inducing the rapid and direct killing of activated HSCs. These mechanisms suggest that iNKT cells may also influence the development of liver fibrosis in humans (52). However, the role of NKT cells in pediatric post-transplant liver fibrosis remains unknown.

### Neutrophils

Neutrophils account for about 50–70% of all circulating leukocytes in human beings, serving as the first line of host defense against microbial infections. Emerging evidence shows that, apart from traditional recognition of neutrophils' ability to kill extracellular pathogens, they are also able to mediate other immune cells such as DCs, NK cells, and lymphocytes. The neutrophils induce a complex cross immune response in intracellular pathogens and viruses by secreting a host of cytokines (such as CCL3, CCL20, B cell-activating factor, a proliferation-inducing ligand) or expressing a huge quantity of cell surface molecules (such as S100A and various antimicrobial peptides), which directly interact with other immune cells (53, 54). As a study released in 2014 has found, neutrophils can amplify NK-derived IFN-γ in inflammatory lesions in Crohn's disease patients during active inflammation by interacting with both DC and NK cells (55). Indeed, many NK cell-derived cytokines, including IFN-γ and GM-CSF, have effects on neutrophils, thus enhancing inflammatory responses, which may influence the development of liver fibrosis.

### Kuppfer Cells

Hepatic macrophages (Kupffer cells) lead a significant role in the pathogenesis of chronic liver injury, including chronic inflammation and liver fibrosis (56). They can regulate the process of liver fibrosis bidirectionally (57). In the early stage, hepatic macrophages promote fibrosis by aggregating pro-inflammatory immune cells and secreting pro-inflammatory cytokines and chemokines, while in the later stage, they propel the regression of liver fibrosis by secreting matrix metalloproteinases (MMPs).

Hepatic macrophages interact with other immune cells. For example, they attract NKT cells by releasing chemokines CXCL16 in the early stage. These immune cells also participate in the progress of liver fibrosis. Meanwhile, NKT cells can activate pro-inflammatory signals in Kuppfer cells (58). Besides, Kuppfer cells activate hepatic stellate cells(HSCs) through a paracrine mechanism. HSCs can then differentiate into myofibroblasts which produce collagen fibers (59, 60).

For those patients who have recurrent hepatitis C disease after liver transplantation, chronic HCV causes an excessive healing response, which leads to the formation of liver fibrosis after liver transplantation. In this process, M1 macrophages, which belong to the proinflammatory cells, may participate in the fibrinolysis process, while cells that inhibit chronic inflammation such as M2 macrophages would produce profibrogenic factors to activate HSCs and myofibroblasts (61).

Other studies have shown that macrophages can cause chronic rejection and fibrosis after kidney transplantation. Steroids and calcineurin inhibitors, which are the routine medicines prescribed after organ transplant surgery, have been shown to induce polarization of CD163 + M2 macrophages, thereby promoting fibrosis and increasing rejection (62, 63). Therefore, we speculate that hepatic macrophages may also participate in the process of liver fibrogenesis after liver transplantation, which may be applied to adult and even pediatric patients, but this conjecture needs to be proven by further research.

## Innate Immunity and Pediatric Post-Transplant Idiopathic Liver Fibrosis

### Impact of Pediatric Immunity Development and Primary Disease Before Transplantation

The immune status in the pediatric population is quite distinctive. Compared with adults, the pediatric immune system is still in development. According to a study of pediatric immune status before liver transplantation (64), the majority of immune cells, except for granulocytes, showed a logarithmic linear decline with age, which may be mainly related to the decline of the early thymus output of T cells, which in turn affects the level of B cells, NK cells, monocytes, and cytokines.

Compared with adults, it was confirmed through a biopsy that recurrence of primary disease is the most common cause of late graft dysfunction in adults, whereas, for children, unexplained idiopathic hepatitis and liver fibrosis are the main causes (65). Among pre-transplant children, serum CD19+ B cell level is the highest in those with biliary atresia, while it is lowest in those with tumors. Meanwhile, the T cell level is highest in children with advanced cancer but lowest in those with acute liver failure, and there is no significant difference in the level of monocytes between these patient groups. This trend of immune cells can also be observed in soluble immune markers. Correspondingly, TH2 cytokines, such as IL-4, IL-5, and IL-13, were selectively increased in pediatric patients with tumors, similar to the change of CCL11, IL-16, and CXCL1 in biliary atresia patients. It can be inferred that the difference in individual diseases also has an impact on immune status. With respect to the relative contribution of these two factors to immunological variance, Tamara et al. found from the ChilsSFree multicenter cohort study that 75% were related to age and 1–16% was attributed to the underlying primary disease, both of which are independent to each other (64). Given the different pre-transplant immunological status in children, the interpretation of immunological surveillance after liver transplants may need to take pre-transplant variables into account.

### Modulation of Immunosuppressive Therapy on Anti-fibrosis Effect of Innate Immunity

The use of Tacrolimus (FK506) is widespread in the field of pediatric liver transplantations in regulating post-surgery rejection. As the first-line immunosuppressive treatment, the dose is tapered and adjusted individually, geared to achieve a balance of anti-rejection and anti-fibrosis, efficacy, and minimizing toxicity. It is a macrolide immunosuppressive agent that has a strong immunosuppressive function. Vitro and animal experiments have proven that tacrolimus can inhibit the development of fibrosis (66, 67). Clinical reports have also suggested that tacrolimus can effectively inhibit calcineurin (CN) activity and cytokine production, allowing better immune tolerance in the liver of patients post-transplant (68). This is further verified in another article, which outlines that the inadequate use of tacrolimus after living-donor LT in pediatric patients can cause post-operative centrilobular fibrosis (8).

However, FK506 has also been proven to have the ability to inhibit NK cells, which play a vital role in inhibiting fibrogenesis, *in vitro* to a significant extent. Tae-Jin Kim has suggested that FK506 can directly contribute to defective NK cell clustering accompanied by down-regulation of the cell adhesion molecules, ICAM-1, CD2, CD49d, and CD58. It can also selectively down-regulate NK activating receptors, inhibiting the expression of NKG2D, CD48, and DNAM1 receptors without affecting 2B4, NKp30, NKp44, and NKp46 (69). Furthermore, FK506-treated NK cells are reported to have impaired IL-2R signaling and STAT3 inhibition, which is one of the research hotspots in fibrosis. The impairment of NK cell activation, enrollment, and protective signals caused by tacrolimus also has a possible influence on the genesis of pediatric idiopathic fibrosis after allograft liver transplantation.

**Figure 2:**
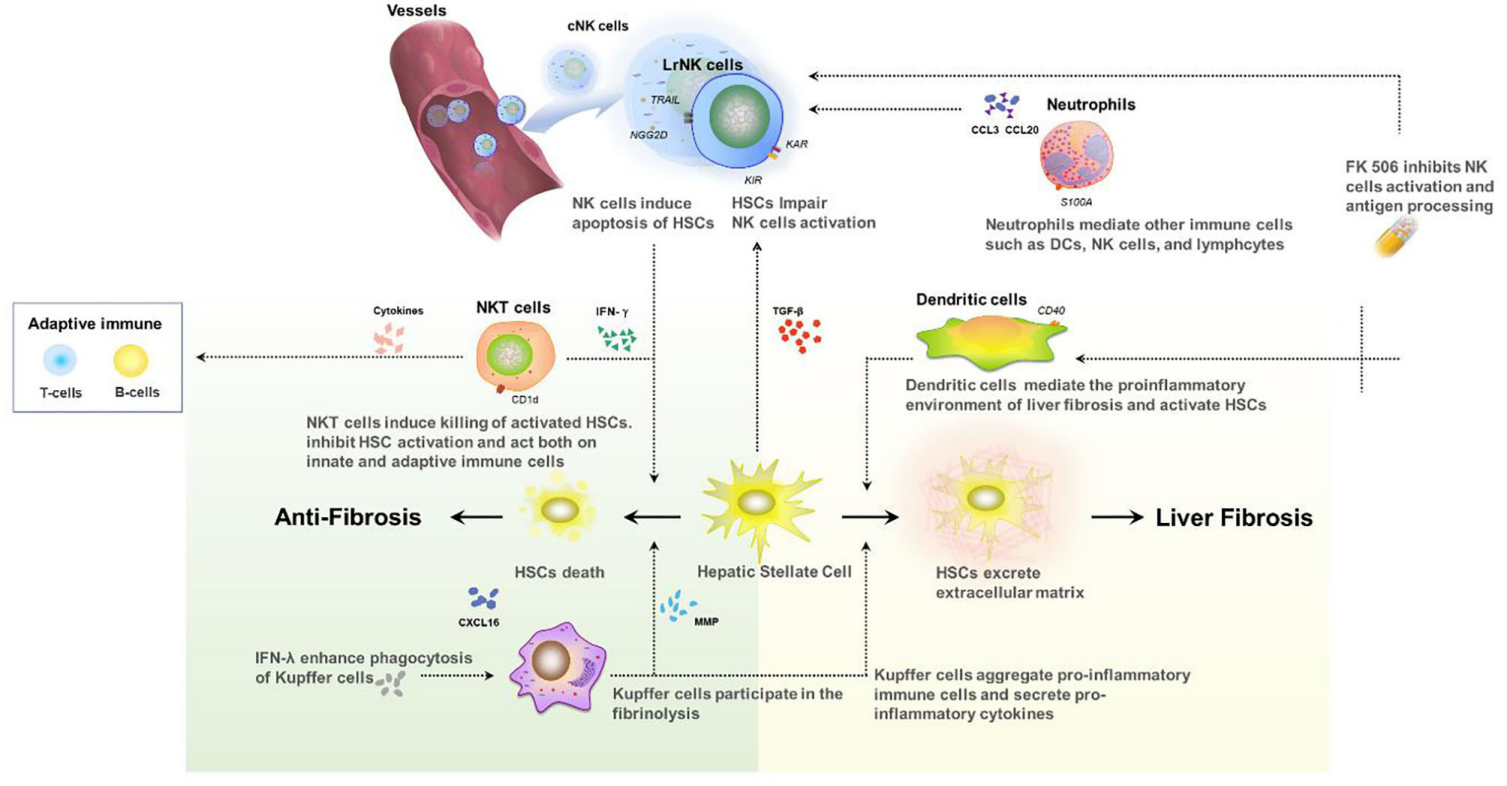
Interaction between innate immune cells in the development of liver fibrosis after transplant.

It should be noted that hepatic fibrosis in pediatric recipients is a complex process involving multiple immune cells (Figure 2). Although the reduction of NK cells and interferon can promote graft survival, cytotoxicity in the early-activated HSC cells can be also impaired, which may accelerate fibrosis. Only a few studies have focused on the relationship of immunosuppressive therapy and the long-term development of pediatric post-transplant idiopathic liver fibrosis, and there is little information on the underlying mechanisms. Further research is needed to address the molecular mechanism of periportal, centrilobular, and perisinusoidal in pediatric patients with FK506 or other immunosuppressive therapy.

## Conclusion and Perspectives

To date, multiple studies have proven that innate immunity plays a key role in the development of liver fibrosis. As described in this review, the following immune cells and components have participated in liver fibrosis: IFN-λ is considered to stimulate liver fibrosis whereas both IFN-α/β and IFN-γ inhibit this procedure. TLRs participate in the development of liver fibrosis after transplantation. Liver dendritic cells regulate inflammation and fibrosis in the liver microenvironment. Kupffer cells stimulate liver fibrosis whereas neutrophils seem to have less effect. NK cells inhibit liver fibrosis by killing activated HSCs and IFN-γ production, while the role of NKT cells during fibrosis remains ambiguous. Meanwhile, tacrolimus therapy after LT plays a complex role in the development of post-operative idiopathic liver fibrosis. On the one hand, such therapy allows better immune tolerance, which can have some anti-fibrosis effect for pediatric patients. One the other hand, FK506 can inhibit NK cells, which may accelerate fibrosis. Due to the unique immune status of pediatric patients and specific graft tolerance, the immune mechanism of pediatric post-transplant liver fibrosis remains unknown. Understanding this underlying mechanism will provide new theoretical thinking on the use of immunosuppressive agents in children after liver transplantation. Innate immunity, therefore, is an important therapeutic target in the prevention and reduction of long-term idiopathic liver fibrosis, which could improve the long-term survival rate and outcomes of liver transplants. Further studies are required to clarify the role that the main immune components play in post-transplant liver fibrosis in children of different age groups as well as the specific influence of immunosuppressive agents and immune cells on this procedure.

## Author Contributions

All authors listed have made a substantial, direct and intellectual contribution to the work, and approved it for publication.

## Conflict of Interest

The authors declare that the research was conducted in the absence of any commercial or financial relationships that could be construed as a potential conflict of interest.
